# Computational Metabolomics Operations at BioCyc.org

**DOI:** 10.3390/metabo5020291

**Published:** 2015-05-22

**Authors:** Peter D. Karp, Richard Billington, Timothy A. Holland, Anamika Kothari, Markus Krummenacker, Daniel Weaver, Mario Latendresse, Suzanne Paley

**Affiliations:** Bioinformatics Research Group, SRI International, 333 Ravenswood Ave AE206, Menlo Park, CA 94025, USA; E-Mails: billingt@ai.sri.com (R.B.); timothy.holland@sri.com (T.A.H.); kothari@ai.sri.com (A.K.); kr@ai.sri.com (M.K.); weaver@ai.sri.com (D.W.); latendre@ai.sri.com (M.L.); paley@ai.sri.com (S.P.)

**Keywords:** metabolic pathways, metabolomics data analysis, metabolic database

## Abstract

BioCyc.org is a genome and metabolic pathway web portal covering 5500 organisms, including *Homo sapiens*, *Arabidopsis thaliana*, *Saccharomyces cerevisiae* and *Escherichia coli*. These organism-specific databases have undergone variable degrees of curation. The EcoCyc (*Escherichia coli* Encyclopedia) database is the most highly curated; its contents have been derived from 27,000 publications. The MetaCyc (Metabolic Encyclopedia) database within BioCyc is a “universal” metabolic database that describes pathways, reactions, enzymes and metabolites from all domains of life. Metabolic pathways provide an organizing framework for analyzing metabolomics data, and the BioCyc website provides computational operations for metabolomics data that include metabolite search and translation of metabolite identifiers across multiple metabolite databases. The site allows researchers to store and manipulate metabolite lists using a facility called SmartTables, which supports metabolite enrichment analysis. That analysis operation identifies metabolite sets that are statistically over-represented for the substrates of specific metabolic pathways. BioCyc also enables visualization of metabolomics data on individual pathway diagrams and on the organism-specific metabolic map diagrams that are available for every BioCyc organism. Most of these operations are available both interactively and as programmatic web services.

## 1. Introduction

Metabolic pathways provide an organizing framework for understanding metabolomics data. The BioCyc.org website provides a number of metabolomics data analysis and visualization services in concert with the 5500 metabolic pathway databases available at the site. These operations include metabolite search, metabolite enrichment analysis, visualization of metabolomics data on pathway diagrams and metabolic map diagrams and translation of metabolite identifiers across multiple metabolite databases. These operations are provided via two access modalities: interactive website operations and programmatic web services. (Note that we use the term “web service” somewhat loosely here to refer both to web APIs (application program interface, the program-callable web services) that generate data and to web APIs that generate web pages.)

BioCyc is a genome and metabolic pathway web portal [1] that contains thousands of pathway/genome databases (PGDBs). Each PGDB describes the genome and metabolic network of a sequenced organism. The biological objects modeled within a PGDB include replicons, genes, proteins, biochemical reactions, metabolites and pathways. For most PGDBs, the metabolic network was inferred computationally by the Pathway Tools software [2,3]. Some PGDBs contain information from extensive manual curation of the biomedical literature, e.g., the contents of the EcoCyc [4] database were derived from more than 27,000 publications.

The MetaCyc [1,5,6] database plays a special role in the BioCyc collection because it is a “universal” metabolic database that describes 2310 pathways, 12,377 reactions, 10,298 enzymes and 11,987 metabolites from all domains of life. The contents of MetaCyc have been derived from 45,000 publications. The vast majority of reactions, metabolites and pathways in the organism-specific PGDBs are a subset of those in MetaCyc. MetaCyc also contains extensive atom mapping data that can be used to track specific atoms through a sequence of metabolic reactions [7].

PGDB data can be accessed in a variety of forms [8]. The BioCyc website provides interactive querying and visualization of these data, as does the downloadable Pathway Tools software bundled with BioCyc databases. BioCyc data can be downloaded as a series of data files, can be queried via web services [9] and can be queried via APIs in Java [10,11], Perl [12], Python [13] and Common Lisp [14].

### Related Work

Our metabolite translation service was inspired by previous work, such as the Chemical Translation Service (CTS) of Wohlgemuth [15], UniChem [16] and MetMask [17]. Rather than translating a single specified type of identifier to another single identifier, as CTS does, our interface allows users to identify each metabolite in one line using as many or as few names and identifiers as they have available, and we indicate all of the identifiers that we have available in BioCyc, again in one line. Our service spans a smaller number of databases than do CTS or UniChem. Our service is not based on automatically-calculated correspondences using InChIs (International Chemical Identifiers) as is UniChem’s, but instead, is based on correspondences stored within each BioCyc database, thus allowing curation of correspondences that are not detected by InChI matching.

KEGG provides metabolomics visualization services for its pathway diagrams via the KEGG Mapper [18]. The KEGG Mapper is similar to the single-data-point tool we describe in Section 2.7.3, but KEGG Mapper cannot depict multiple data points, as Pathway Tools shows via pop-up windows (see Figure 4). It is also important to keep in mind the general differences between KEGG maps and BioCyc pathways discussed in a recent comparison [6]: KEGG maps tend to be significantly larger than BioCyc pathways, because KEGG maps are mosaics of multiple biological pathways across many species, whereas BioCyc pathways are single metabolic pathways found in specific organisms. For example, KEGG map MAP00270 combines individual pathways involved in biosynthesis and degradation of L-cysteine and L-methionine; therefore, metabolite matches to this KEGG map could be confused with several pathways.

KEGG Atlas [19] is an analog of the BioCyc Cellular Overview diagrams. Its stated goal is to support “the mapping of high-throughput experimental data onto the global [metabolic] map.” However, we found KEGG Atlas unable to successfully visualize metabolomics data (specifically, from the web page http://www.kegg.jp/kegg/atlas/?01100, we clicked “eco” at the upper-left to display the diagram, then clicked the “P” button at the top-right and entered several metabolite-ID/color pairs, as per the instructions, but no metabolites were ever highlighted upon clicking “apply”). Furthermore, KEGG Atlas uses a set of seven fixed diagrams for different areas of small-molecule metabolism—fixed in the sense that the same seven diagrams are used for all of the organisms in KEGG. Thus, no one diagram contains all metabolic pathways for all organisms, and many of the reactions in any given diagram are not actually present in any specific organism (reactions not present in a selected organism are grayed out). In contrast, each organism in BioCyc has its own custom-generated Cellular Overview diagram containing only those reactions present in the PGDB for that organism. It does not support animation nor omics pop-up diagrams.

KaPPA-View enables viewing of metabolomics data on individual pathway diagrams for a number of plant species [20]. It does not support viewing on full metabolic network diagrams.

Metscape [21] supports viewing of metabolomics data on diagrams derived from the EHMN metabolic database [22] for individual pathways or for large networks. Diagrams are displayed by Cytoscape [23]. It does support animation, but does not support omics pop-ups. Cytoscape diagrams can be very densely connected, interfering with readability, and do not use graphical conventions typically used for metabolic pathways.

## 2. Results and Discussion

This section presents the metabolomics operations available from the BioCyc website and from the downloadable Pathway Tools software, which includes BioCyc. Furthermore, these operations are available from other Pathway Tools-based websites [24], such as PlantCyc [25] and SolCyc [26] (the services available depend on the version of Pathway Tools installed at a site). We note that the BioCyc operations described herein are processed in the context of a specific PGDB. For example, when a user performs a metabolite search, that operation searches through all metabolites within a specified PGDB (usually the currently selected PGDB), such as the EcoCyc PGDB for *Escherichia coli*. Similarly, when visualizing metabolomics data on a metabolic map diagram, each PGDB has a different metabolic map diagram, and the operation must use the diagram for a specified organism. Additional information about these operations can be found at [27]; online tutorials describing many of these operations can be found at [28].

### 2.1. Metabolite Search

BioCyc supports interactive metabolite searches and metabolite searches via web services.

### 2.2. Metabolite Search: Interactive

The interactive metabolite search enables users to search within a given BioCyc PGDB for metabolites that satisfy one or more criteria. The resulting BioCyc metabolite pages describe chemical properties of the metabolite, as well as the reaction(s) and pathway(s) in which the metabolite participates and the genes and enzymes regulated by the metabolite. This search is available under the website command **Search** → **Search Compounds**. The user can specify combinations of the following search criteria.

- The common name or synonyms of the metabolite must match one or more names or substrings supplied by the user;
- The metabolite contains a database identifier supplied by the user, for databases, including BioCyc, ChEBI [29], KEGG, HMDB [30], and PubChem (see Table 1 for a list of supported external databases);
- The metabolite matches within a specified tolerance of a molecular weight or monoisotopic molecular weight supplied by the user;
- The metabolite matches a full or partial chemical formula supplied by the user;
- The metabolite matches an InChI (specifically, InChI-1S) string supplied by the user;
- The metabolite is within a region of the BioCyc chemical ontology (e.g., “a prostaglandin” or “a steroid”).

### 2.3. Metabolite Search: Web Services

BioCyc provides web services for searching for metabolites based on monoisotopic molecular weight, chemical formula and external database identifiers. It also provides a service for translating metabolite names and identifiers among multiple databases.

#### 2.3.1. Web Service for Search by Monoisotopic Molecular Weight

BioCyc metabolites can be retrieved based on matches to a specified monoisotopic molecular weight and tolerance. This web service can be used if the user is retrieving the results by either the “POST” or “GET” method. The URLs for monoisotopic molecular weight search are as follows.


http://websvc.biocyc.org/[ORGID]/monoisotopicwt?wts=[MONOISOTOPICMW]&tol=[TOLERANCE]
or
http://websvc.biocyc.org/[ORGID]/monoisotopicwt?wts=[MONOISOTOPICMW]&tol=[TOLERANCE]&fmt=json


where:

- [ORGID] is the BioCyc identifier for the organism database, e.g., ECOLI (for EcoCyc), META (MetaCyc), AFER243159 (*Acidithiobacillus ferrooxidans,* Strain ATCC 23270).
- [MONOISOTOPICMW] is a floating point number, standing for the monoisotopic molecular weight in Daltons. One or more monoisotopic molecular weights can be supplied; multiple values are separated by commas.
- [TOLERANCE] is the search tolerance +/− in ppm.
- fmt=json requests output in the JavaScript Object Notation (JSON) format; the default output is in a tab-delimited format.

The JSON format (shown in the last example below and defined at www.json.org) is particularly useful, because JSON data are structured and regular yet easy to parse. JSON is a native format within the JavaScript programming language that is often used to implement web services in a browser, thus JSON can be directly used by JavaScript without any need for parsing.

Example URLs:

- http://websvc.biocyc.org/ECOLI/monoisotopicwt?wts=240.063,88.052&tol=5 Retrieve the compounds that have a monoisotopic molecular weight of either 240.063 or 88.052 with a tolerance of 5 ppm in EcoCyc.
- http://websvc.biocyc.org/BSUB/monoisotopicwt?wts=169.988&tol=5&fmt=json Retrieve the compounds that have a monoisotopic molecular weight of 169.988 within a tolerance of 5 ppm in *Bacillus subtilis* in the JSON format.
- http://websvc.biocyc.org/META/monoisotopicwt?wts=123.009,56&tol=5 Retrieve the compounds that have monoisotopic molecular weights of 123.009 or 56 with a tolerance of 5 ppm in MetaCyc. For 123.009, this query retrieves any compound that has a molecular weight in the range 123.008385 to 123.00962 Daltons. The query results are shown below.

The default output is a tab-delimited format as follows (these results were generated by the preceding example):


123.009 1 123.008705 3-chloro-L-alanine CHLORALAN-CPD
123.009 1 123.008705 3-chloro-D-alanine 3-CHLORO-D-ALANINE
123.009 1 123.008705 2-chloro-L-alanine CPD0-1475
123.009 1 123.008705 3-chloro-DL-alanine 3-CHLORO-DL-ALANINE
56 0


The first column contains the input monoisotopic molecular weights. The second column indicates whether the query was successful (1) or unsuccessful (0). The third column contains the monoisotopic molecular weight of the compound that is stored in the PGDB. The fourth column contains the metabolite name. The fifth column contains the BioCyc identifier of the compound.

JSON format:

 [{“INPUT”:”123.009”,”STATUS”:1,  “RESULTS”:[{“MW”:123.008705,”NAME”:”3-chloro-L-alanine”,”ID”:”CHLORALAN-CPD”},        {“MW”:123.008705,”NAME”:”3-chloro-D-alanine”,”ID”:”3-CHLORO-D-ALANINE”},        {“MW”:123.008705,”NAME”:”2-chloro-L-alanine”,”ID”:”CPD0-1475”},        {“MW”:123.008705,”NAME”:”3-chloro-DL-alanine”,”ID”:”3-CHLORO-DL-ALANINE”}>,
 {“INPUT”:”56”,”STATUS”:0}]


#### 2.3.2. Web Service for Search by Chemical Formula

This service finds the BioCyc IDs of all metabolites that exactly match a supplied chemical formula. This web service can be used if the user is retrieving the results by either the “POST” or “GET” method. The URLs to search metabolites based on chemical formula are as follows.


http://websvc.biocyc.org/[ORGID]/CF?cfs=[CHEMICAL-FORMULA]
or
http://websvc.biocyc.org/[ORGID]/CF?cfs=[CHEMICAL-FORMULA]&fmt=json


where:

- [ORGID] is the identifier for the organism database, e.g., ECOLI, META, AFER243159.
- [CHEMICAL-FORMULA] is the chemical formula to be matched, in the format ([element-symbol][coefficient])+, where “+” is a regular-expression operator that means the preceding expression can be repeated one or more times. The element symbol is case sensitive. Coefficients of one may be omitted.
  The chemical formula input may contain one or more values, which may be separated by commas. The search will return all metabolites in the specified database that have a chemical formula(s) equal to the one provided. Note that two chemical formulas are equal if and only if they are element-wise equal. For example, C6H6 is equal to H6C6 because both have elements H6 and C6.
- fmt=json requests output in JSON format; the default output is in tab-delimited format.

Example URLs:

- http://websvc.biocyc.org/META/CF?cfs=C6H6
  Retrieve the compound that contains a chemical formula of C6H6 in MetaCyc. The result of this search is:

C6H6 1 BENZENE benzene
- http://websvc.biocyc.org/META/CF?cfs=C12H16N3O2S,C6H2N3O3Cl,H3O3
  Retrieve the compounds that match any of the three provided chemical formulas in MetaCyc. The result of this search is:

C12H16N3O2S 1 CPD-15776 oxythiamine
C6H2N3O3Cl 1 CPD0-1250 4-chloro-7-nitrobenzo-2-oxa-1,3-diazole
H3O3 0
- http://websvc.biocyc.org/BSUB/CF?cfs=C6H6&fmt=json
  Retrieve the compound that contains a chemical formula of C6H6 in JSON format. The result of this search is:

[{“INPUT”:”C6H6”,”STATUS”:0}]

The default output is a tab-delimited format, as shown in the second example. The first column contains the provided chemical formula. The second column indicates whether a valid compound that matches the chemical formula was found (1). The third column contains the BioCyc identifier of the compound that matches the chemical formula. The fourth column contains the common-name of the compound.

#### 2.3.3. Web Service for Search by an External Database Identifier

This service finds the BioCyc ID of a metabolite given a foreign ID, that is an identifier of that object in an external database. This service depends on the foreign ID being stored in the DB-Links slot of the BioCyc metabolite, and we note that BioCyc’s links to external databases are incomplete. This search works not only for metabolites, but also for BioCyc objects, such as pathways, reactions, genes and proteins. This web service can be used if the user is retrieving the results by either the “POST” or “GET” method. URLs to search for metabolites based on the foreign identifier are as follows.


\http://websvc.biocyc.org/[ORGID]/foreignid?ids=[DATABASE-NAME]:[FOREIGNID]
or
http://websvc.biocyc.org/[ORGID]/foreignid?ids=[DATABASE-NAME]:[FOREIGNID]&fmt=json


where:

- [ORGID] is the identifier for the organism database, e.g., ECOLI, META, AFER243159.
- [DATABASE-NAME] is the name of external database, e.g., KEGG, PUBCHEM, UNIPROT, and is case-insensitive.
- [FOREIGNID] is the external database identifier. The foreign id input may contain one or more values, which may be separated by commas. Foreign IDs are case sensitive.
- fmt=json requests output in the JSON format; the default output is in tab-delimited format.

Example:

- http://websvc.biocyc.org/META/foreignid?ids=Kegg:C00849
  Retrieve the MetaCyc metabolite that contains a KEGG ID of C00849.

The default output is a tab-delimited format as follows:


Kegg:C00849 1 ETHYLACETATE


The first column is the input foreign ID. The second column indicates whether a valid object that matches the foreign ID was found (1). The third column indicates the BioCyc identifier of the object that matches the foreign ID.

### 2.4. Metabolite Translation Service

BioCyc provides both interactive and programmatic services to translate sets of metabolites specified in many alternative ways to the BioCyc identifiers for those metabolites and to identifiers for external databases. For example, a user could supply a list of KEGG or PubChem metabolite identifiers, or a list of InChI-1S strings, or a list of chemical names, and obtain the BioCyc and ChEBI identifiers for all of the preceding items that are recognized. This service can be invoked interactively through [31].

The input to the service is a set of lines, one line per metabolite. Each line contains one or more metabolite names, identifiers and an optional InChI string, InChI key, monoisotopic molecular weight and chemical formula. The service looks up each of the preceding fields within the specified BioCyc database. Three cases are possible for each line:

(1) None of the provided fields is recognized in the specified BioCyc database, in which case “unknown” is returned, along with the unknown input fields.
(2) All of the recognized names, identifiers, InChI string, InChI key, monoisotopic weight and chemical formula match a single metabolite, in which case “successful” is returned, along with the following tab-separated fields:
  - BioCyc ID of the matching metabolite;
  - BioCyc common name of the matching metabolite;
  - Additional identifiers present in BioCyc from other databases for the matching metabolite ;
  - InChI-1S string for the metabolite.
  In this case, unrecognized input items are ignored.
(3) The recognized names, identifiers, InChI string, InChI key, monoisotopic weight and chemical formula match more than one BioCyc metabolite, in which case “ambiguous” is returned, along with the multiple matches that were found.

Consider the following example (note that the line number prefixes, such as L1, were added here to facilitate understanding and should not be provided in the input data).

Input:


L1: PubChem:125 Kegg:C00001
L2: InChI:InChI=1S/C2H4O2/c1-2(3)4/h1H3,(H,3,4)/p-1
L3: CF:C6H6 MW:78.0469501 InChI-key:InChIKey=UHOVQNZJYSORNB-UHFFFAOYSA-N
L4: TRP
L5: Kegg:C00183 InChI:InChI=1S/C5H11NO2/c1-3(2)4(6)5(7)8/h3-4H,6H2,1-2H3,(H,7,8)/t4-/m0/s1
L6: Kegg:C00004
L7: Kegg:C45698
L8: Kegg:C00001 MW:18.01056468


Output (InChI strings are omitted from the output for brevity):


L1: ambiguous BioCyc:4-HYDROXY-BENZYL-ALCOHOL BioCyc:WATER
L2: success BioCyc:ACET acetate HMDB:HMDB00042 DrugBank:DB03166 IAF1260:33590 ChemSpider:170 PubChem:175 ChEBI:30089 KEGG:C00033
L3: success BioCyc:BENZENE benzene HMDB:HMDB01505 ChemSpider:236 PubChem:241 ChEBI:16716 KEGG:C01407
L4: success BioCyc:TRP L-tryptophan HMDB:HMDB00929 IAF1260:33772 ChEBI:57912 PubChem:6923516 KEGG:C00078
L5: success BioCyc:VAL L-valine MetaboLights:MTBLC57762 HMDB:HMDB00883 IAF1260:34167 ChEBI:57762 PubChem:6971018 KEGG:C00183 CAS:72-18-4
L6: success BioCyc:NADH NADH HMDB:HMDB01487 IAF1260:33484 ChemSpider:10239197 ChEBI:57945 PubChem:21604869 KEGG:C00004
L7: unknown Kegg:C45698
L8: ambiguous BioCyc:WATER BioCyc:OH


### 2.5. Manipulating Metabolite Sets with SmartTables

The SmartTables facility in BioCyc (formerly called “Web Groups” [32]) allows users to define, store, manipulate and share lists of metabolites, genes, pathways and other biological entities. SmartTables are analogous to spreadsheets in that they can display multiple columns of information about a set of metabolites. However, rather than being supplied by the user, typically, some columns of a SmartTable are derived from a BioCyc database. SmartTables can be used to drive some of the analyses described in later sections, such as metabolite enrichment analysis. In our experience, SmartTables are easy for biologists to use, and provide powerful analyses that previously would have required the assistance of a programmer.

SmartTable operations are found under the SmartTables item in the main BioCyc menu. Because SmartTables are associated with a user’s BioCyc account, it is best to create a BioCyc account before working with SmartTables.

**Figure 1:**
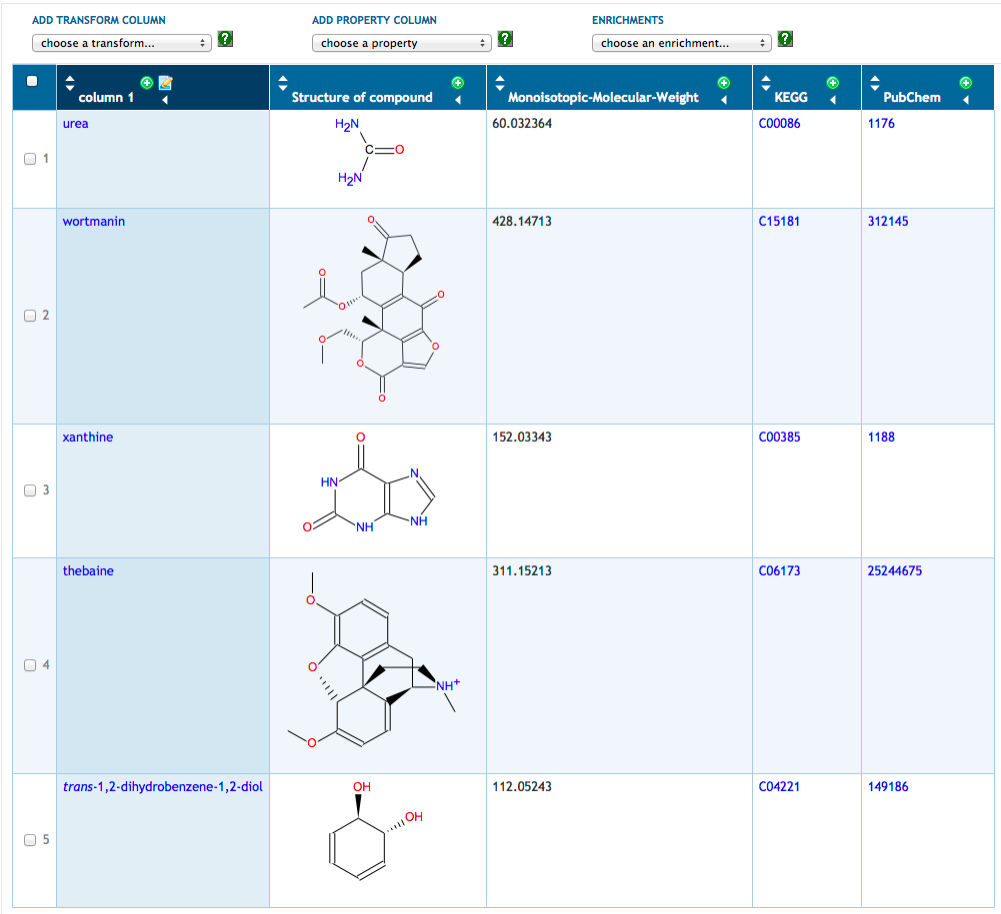
Metabolite SmartTable containing 125 metabolites from Chen, 2012; the first five metabolites are shown here because of space limitations.

A typical scenario is to for a user to define a new SmartTable by importing a file of metabolites. The metabolites are specified by name, by BioCyc ID or by external database ID. To illustrate SmartTables, we have created a SmartTable containing metabolomics data from a personal omics study [33] (see Figure 1). This SmartTable contains the metabolites observed during the second infection period reported in this study. We manually mapped the monoisotopic masses provided as supplementary data to HumanCyc metabolite identifiers. Multiple matches were discarded. We took the union of the resulting positive-mode and negative-mode metabolite sets. We imported a file of these metabolite identifiers to construct the SmartTable, which can be accessed through http://humancyc.org/group?id=biocyc13-61-3633296894.

Once the SmartTable is defined, the user can display database attributes of the metabolites as SmartTable columns through the “Add Property Column” menu. In Figure 1, we have added columns showing the chemical structure, monoisotopic molecular weight and identifiers from KEGG and PubChem. Other operations available for SmartTables include combining two SmartTables through set operations (e.g., take the union of the metabolites in two SmartTables), filtering a SmartTable to retain or remove those metabolites that meet some criterion (e.g., remove from the SmartTable those metabolites with a monoisotopic molecular weight greater than 500) and exporting the contents of a SmartTable to a file.

**Figure 2:**
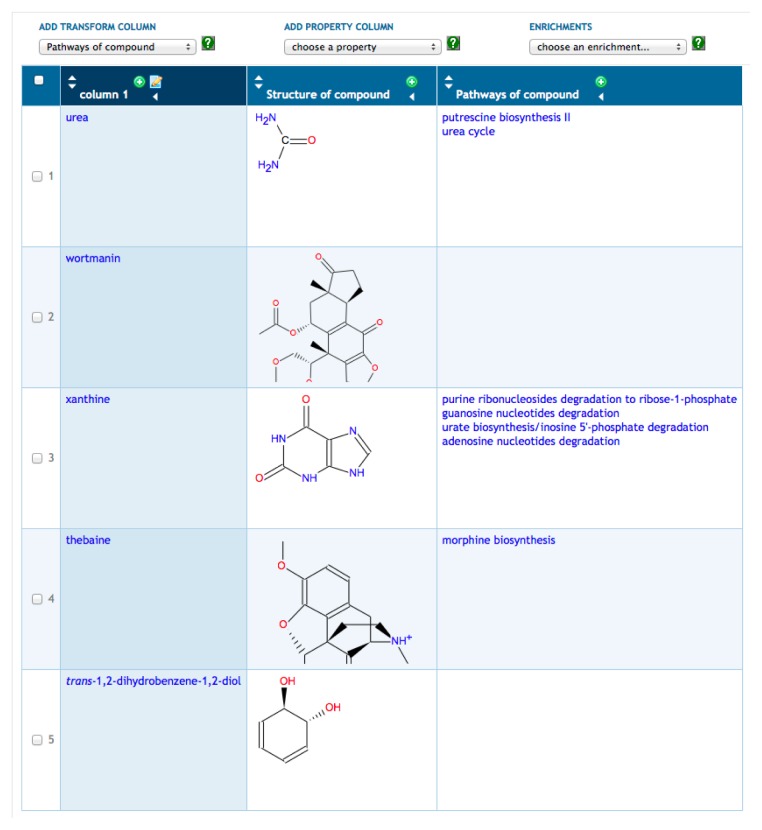
This metabolite SmartTable was derived from the SmartTable in Figure 1 by adding Column 3, which transforms the metabolites in Column 1 to the metabolic pathways containing those metabolites.

Transformations are a more advanced set of SmartTable operations that compute a new column of information for a SmartTable based on a starting column. For example, in Figure 2, we have transformed our metabolite SmartTable by adding a column that lists all metabolic pathways in which each metabolite from Column 1 is a substrate within that PGDB. Other transformations available for metabolites include computing the set of reactions that a metabolite is involved in, computing the set of genes or enzymes regulated by the metabolite and computing the set of proteins known to bind the metabolite. Given a new column created by a transformation, clicking on the “+” at the top of the column will create a new SmartTable containing all objects in that column (*i.e.*, the set of pathways in Figure 2).

Different sets of transformations are available for different types of SmartTables. For example, a SmartTable of pathways can be transformed into the set of genes in the pathway, or the set of reactions in the pathway, or the set of metabolites in the pathway. A SmartTable of genes can be transformed into those genes that regulate the initial gene set, into those genes that are regulated by the initial gene set, into those genes that are present (as orthologs) in another PGDB, or into the metabolic pathways in which each initial gene plays a role.

### 2.6. Metabolite Enrichment Analysis

When analyzing a metabolomics dataset, one question that arises is whether the list of metabolites is over-represented for metabolites in particular metabolic pathways. That is, does the list of metabolites contain more compounds from certain metabolic pathways than one would expect by chance? This question can be answered using Fisher’s exact test of statistical significance. Because different pathways contain different numbers of metabolites, the probability of observing a metabolite by chance differs for different pathways.

**Figure 3:**
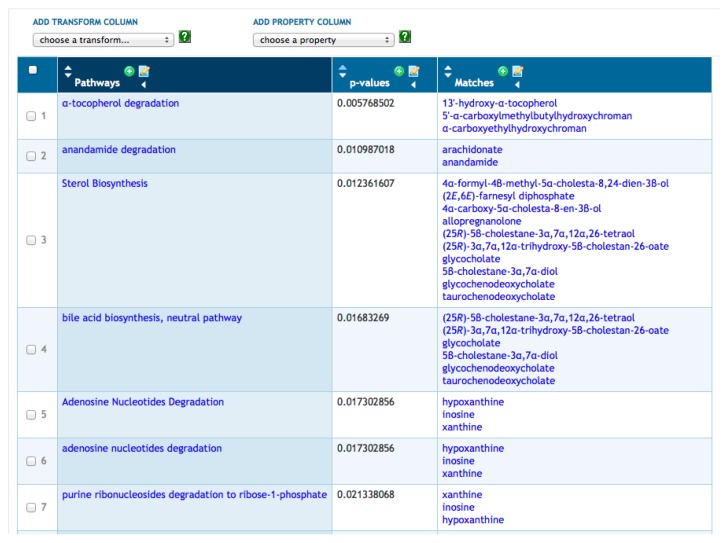
SmartTable containing results of running a metabolic pathway enrichment analysis on the metabolite SmartTable in Figure 1.

BioCyc provides metabolite enrichment analysis via SmartTables. Given a SmartTable of metabolites (e.g., Figure 1), the Enrichments menu allows the user to compute pathway enrichment analysis on that metabolite set using three variations of the Fisher exact test and using four possible multiple-testing corrections. The operation results in a new SmartTable (see Figure 3); its first column contains every pathway containing at least one metabolite from the starting SmartTable; its second column contains a *p*-value indicating the probability of finding the observed metabolites from that pathway by chance; and its third column lists those metabolites from the starting SmartTable that are present in each pathway.

### 2.7. Metabolite Visualization on Pathway Diagrams

BioCyc provides both an interactive tool for painting metabolomics data onto pathway diagrams and a web-service interface to this facility. Both approaches provide several possible display formats, which depend in part on whether a single data point (e.g., a concentration) is provided for each metabolite or whether multiple data points are provided:

- Single data point: In this case, the supplied data values are depicted as colored boxes next to each metabolite in the pathway diagram, with the colors indicating the data values.
- Multiple data points: In this case, the metabolite data are depicted within individual pop-up window, one window per metabolite. These windows can be formatted as vertical bar charts, heat maps or X-Y plots.

In both approaches, the metabolomics data are provided in a tab-delimited data file. We next describe that file format.

#### 2.7.1. Metabolomics Data File Format

The data-file format for supplying metabolomics data is as follows. The first column of each line specifies a metabolite, and some subsequent tab-separated columns specify metabolomics data values. Those data values can represent concentrations or any other quantity and can result from any processing chosen by the user. Note that not every data column need contain metabolomics data; the user selects which columns will be used in the visualizations, and all other columns are ignored.

An excerpt from a sample data file follows (lines beginning with the “#” character are ignored):


#
# Example metabolomics data file
#
1-hydroxy-2-propanone -8.85 6.72 1.65 5.12
BETAINE_ALDEHYDE 8.03 -6.06 5.13 1.4
ChEBI:16947 1.26 5.39 -9.71 9.8
PubChem:68107 2.05 2.93 4.12 -5.78
glutarate semialdehyde$BioCyc:CPD-280$KEGG:C03273 8.81 4.59 6.45 9.46
ChemSpider:131491$HMDB:HMDB$01164 -5.79 1.58 5.84 8.53


Because of the many alternative names and database identifiers used by different metabolomics databases and software systems, we provide a flexible mechanism for specifying metabolites. The user can specify each metabolite in one or more names or identifiers, and the software will attempt to recognize the metabolite using all of the information provided. The first data line in the preceding example shows a metabolite specified by a chemical name known to the HumanCyc database. The second line shows a metabolite specified by the BioCyc identifier used for the compound (in the vast majority of cases, the same identifier is used for a given metabolite in every BioCyc database). In the third and fourth lines, metabolites are specified using their ChEBI and PubChem identifiers, respectively.

The fifth and sixth lines show examples of specifying a single metabolite using multiple alternatives separated by the “$” character; in the case of Line 5, a chemical name, BioCyc ID and KEGG ID are provided. When multiple alternatives are provided, the software ignores alternatives that are unknown to it. The software reports when the alternatives provided in one line identify different metabolites.

#### 2.7.2. Metabolite Visualization on Pathways: Interactive

**Figure 4:**
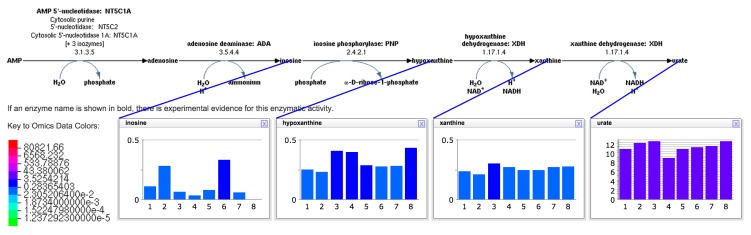
HumanCyc adenosine nucleotide degradation pathway (SALVADEHYPOX-PWY) overlayed with metabolomics data. The data for each metabolite are shown in a separate “omics pop-up” window with eight time points provided for each metabolite. Data were derived from metabolomics data of Chen *et al.* acquired during the second infection period (diabetic onset and respiratory syncytial virus infection). Data shown represent the daily mean of negative-mode LC-MS peak intensities computed from observed compound peaks in three technical replicates and scaled by a factor of 1e-6.

The interactive tool for visualizing metabolomics data on pathways can be invoked by first retrieving a pathway page at BioCyc (example: [34]) and then selecting the right-sidebar command “Customize or Overlay Omics Data on Pathway Diagram.” The resulting pop-up window enables customization of the pathway diagram by designating which elements of the diagram should be present, by overlaying omics data onto the diagram via an imported data file, with the result such as that shown in Figure 4.

#### 2.7.3. Metabolite Visualization on Pathways: Web Service

The visualization shown in Figure 4 can be produced by opening an appropriately formatted URL, thus allowing external software packages to display metabolomics data onto BioCyc pathway diagrams. The URL specifies a pathway to display, formatting parameters for the pathway, the omics dataset to overlay on the pathways and parameters for that overlay operation. The URL in fact generates a larger web page containing both the pathway diagram and accompanying information, such as a pathway mini-review and literature citations. URLs to generate a pathway web page with overlayed omics data are of the form:


http://biocyc.org/[ORGID]/new-image?type=PATHWAY&object=[PATHWAY]&[PARAMETER]=[VALUE]


where:

- [ORGID] is the identifier for the organism database, e.g., ECOLI, META, AFER243159;
- [PATHWAY] is in the BioCyc identifier for the pathway, e.g., GLYCOLYSIS, ARGSYN-PWY, PWY0-1299;
- Multiple parameter/value pairs can be specified (see Table 2).

The omics data can be supplied in two ways: as a POST request (which allows the omics data to be supplied from the local computer) and as a GET request, where a parameter to the GET request is itself a URL that provides the location of the omics data on a publicly accessible web server.

Table 2 explains the parameters that control the overlay of omics data onto pathway diagrams; the parameters in Table 3 control the rendering of the pathway itself.

Additional details regarding Table 2 are as follows:

- *Log* parameter: Applicable when *expressiontype* = *relative*. **On** implies the data are log ratios that use a zero-centered scale—that the numerical data within the omics file may contain positive and negative values. The value zero is considered to be the center of the numerical values provided in the data file.
  **Off** implies the data are ratios that use a one-centered scale—any negative or zero values in the data file will be ignored. For example, the value 0.1 is considered to be at the same distance to one as the value 10. Accordingly, a logarithm of base 10 is applied to the data before the linear coloring mapping is applied.
- *omicsPopups* parameter: Omics pop-up windows are the only way to show data from multiple columns in the input file. A single column of omics data can be shown either in pop-ups or (if unspecified) as color-coded squares within the diagram.
- *Color* parameter: Data values are divided into color bins. This parameter determines which color scale is used—a color scheme that ranges from orange (most positive values) through gray in the center to blue (most negative values); or from red through blue in the center to green and yellow. For each of these color schemes, two options are available:
  – The color bins range over the entire color scale, and the cutoff values for the color bins are derived from the data itself. As a result, different experiments could be displayed using different color schemes, making it difficult to compare them directly.
  – Users may specify a value for the maximum value cutoff (*maxcutoff* parameter) bin. All displays that use the same maximum value cutoff will use the same color scale (assuming other settings are the same) and are therefore directly comparable. All data values greater than the maximum cutoff value will be displayed in the highest bin color.
  – A final alternative is to use only three-color bins, red for data values that exceed some threshold (see the parameter below), purple for data values that are less than the inverse of that threshold and gray for values in between.

### 2.8. Metabolite Visualization on Metabolic Map Diagrams

Pathway-enrichment analysis and pathway visualization of metabolomics data treat metabolomics data in a localized context, that of individual metabolic pathways. In contrast, the operations described in this section enable metabolomics data to be visualized in the context of the full metabolic network of an organism. Each BioCyc PGDB contains an automatically generated diagram called the Cellular Overview. This diagram is an organism-specific view of the metabolic pathways and transporters of the organism. The diagram is zoomable and searchable, can be colored with animated experimental datasets and is generated using the command **Metabolism**→ **Cellular Overview**.

**Figure 5:**
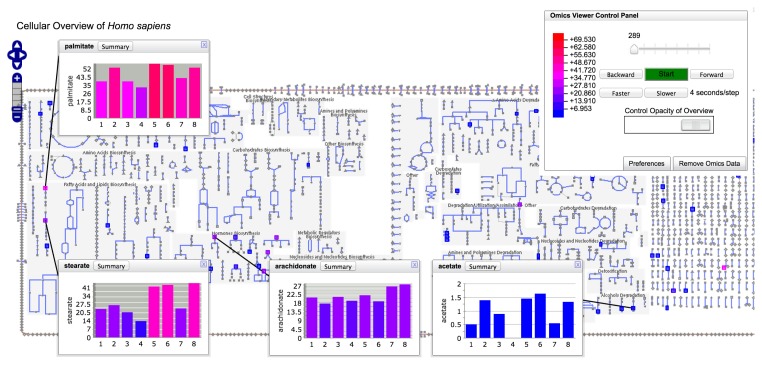
Human Cellular Overview diagram highlighted with metabolomics data. The Omics Viewer Control Panel in the upper right shows the mapping of metabolomics concentration values to colors and allows the user to start, stop and change the speed of the animation. Data were derived from metabolomics data of Chen *et al*. acquired during a period of diabetic onset and respiratory syncytial virus infection. Data shown represent the daily mean of positive-mode LC-MS peak intensities computed from observed compound peaks in three technical replicates and scaled by a factor of 1e-6. The right side of the Cellular Overview has been omitted because of its size.

The diagram (see Figure 5) is bounded by the cell membrane, in which transporters are embedded. The TCA cycle and other energy-generating pathways flow down the middle of the diagram, with biosynthetic pathways to their left and catabolic pathways to their right. Individual reactions not assigned to pathways are in the region to the right of the catabolic pathways. Dots represent metabolites, and lines represent metabolic reactions. Mousing over an element of the diagram will identify it via a tooltip window. In addition, the tooltips provide a button that enables the user to create a visualization of the data for that diagram element in a pop-up similar to those shown in Figure 4. Because providing pop-up-style displays for all of the data mapped to a Cellular Overview would clutter the screen with hundreds of pop-ups, users can create such windows manually. The right-sidebar menu available for the diagram contains multiple commands for searching the diagram, such as identifying metabolites by name or by substring.

Users can create colored overlays of the diagram to aid understanding of experimental datasets, including gene expression, metabolomics and multi-omics datasets. Experimental data are uploaded via a tab-delimited file using the format described in Section 2.7.1. Its first column identifies the entity to be colored; its subsequent columns contain numerical values that determine the coloring. If more than one numerical column is specified for coloring, an animated view is generated in which each time-point of the animation corresponds to one data column. Each frame of the animation shows a different column of data.

## 3. Conclusions

The BioCyc.org website provides a number of operations that allow scientists to analyze metabolomics data in the context of metabolic pathways. Scientists can search for metabolites by chemical formula and monoisotopic molecular weight. The site enables translation of metabolite identifiers across multiple metabolite databases. Researchers can store metabolite lists as SmartTables within their BioCyc account. SmartTables enable exploration of the relationships among metabolites via transformations and set operations, and SmartTables can be shared with selected collaborators or made publicly accessible. SmartTables also support metabolite enrichment analysis, an operation that identifies metabolite sets that are statistically over-represented for the substrates of specific metabolic pathways. BioCyc also enables visualization of metabolomics data on individual pathway diagrams and on the organism-specific metabolic map diagrams that are available for every BioCyc organism. Most of the preceding operations are available both interactively and as programmatic web services.

## Figures and Tables

**Table 1:** The metabolite databases to which BioCyc databases currently contain significant numbers of external links, for which external identifier searches are therefore likely to work; the names for those databases used in BioCyc searches; and the number of links to each database in MetaCyc Version 18.5. That MetaCyc version contains 11,681 metabolites.

Database	Database Name	Link Count
PubChem Compound	PUBCHEM	10,135
ChEBI	CHEBI	6268
KEGG	KEGG	4924
ChemSpider	CHEMSPIDER	4100
DrugBank	DRUGBANK	402
Lipid Maps	LIPID_MAPS	385
HMDB	HMDB	2733

**Table 2:** Parameters to customize pathway omics-data visualizations.

Parameter Name	Possible Values	Description	Default
object	Frame ID, e.g., PWY-7180	The BioCyc unique identifier for a pathway.	***REQUIRED*** no default
datafile	Filename	Name of file containing omics data. Should not be used for a GET.	***REQUIRED*** for POST no default
url	A URL	A URL that points to omics data file. Should not be used for a POST.	***REQUIRED*** for GET no default
class	**gene**, **protein**, **compound**, **reaction**, **NIL**	This parameter declares the type(s) of the IDs found in Column 0 (the first column) of the data file, e.g., **gene** means that Column 0 contains genes. **NIL** means any of the four possible types may be present.	**gene**
datacolumns	numbers and/or ranges of numbers	Specifies data columns from datafile to be painted on pathway graphic	***REQUIRED*** no default
expressiontype	**relative**, **absolute**	**relative**: the numerical data are ratios (e.g., relative to a control) and are therefore centered around either 0 or 1 (see log parameter). **absolute**: the numerical data are absolute values, e.g., intensities or concentrations, and all non-negative.	**relative**
log	**on**, **off**	Indicates whether the provided omics data are log values (**on**) or absolute values (**off**).	**on**
omicspopups	**on** or omitted	**On:** display the omics data in pop-up windows, usually one pop-up per reaction or metabolite.	unspecified
color	**default**, **specify**, **rbg**, **rbg-cutoff**, **3-color**	Selects color scale used to color omics data. **default:** Scale ranges from orange to blue. **specify:** Orange to blue with a maximum cutoff (*maxcutoff* parameter detailed in this table). **rbg:** Red to green. **rbg-cutoff:** Red to green with a maximum cutoff (*maxcutoff* parameter detailed in this table). **3-color:** Three-color display with a threshold.	**default**
threshold	a numeric value	Required if *3-color* specified. See discussion in the “color” parameter section of this table.	no default
maxcutoff	a numeric value	Required if *specify* or *rbg-cutoff* specified. See the discussion in the “color” parameter section of this table.	no default
defaultpopup	**bar**, **plot**, **heat**	The style of data display in the omics pop-ups: a bar graph, an X-Y plot or a heat map.	**bar**

**Table 3:** Parameters to customize pathway appearance.

Parameter Name	Possible Values	Description	Default
detail level	**0**, **1**, **2**, **3**, **4**	Specifies the detail display level for the pathway. The presence of specific elements can be individually determined using other parameters. **0**: Minimal detail, only start/end/branch point metabolites shown. **1**: Show main compounds (those along the pathway backbone). **2**: Show main and side compounds, enzyme and gene names and EC numbers. **3**: Show structures for most main compounds. **4**: Show structures for most main and side compounds.	0, 1 or 2, depending on the size and complexity of the individual pathway.
enz	**y**, **n**	Should enzyme names be shown?	Depends on detail-level.
gene	**y**, **n**	Should gene names be shown?	Depends on detail-level.
ec	**y**, **n**	Should EC numbers be shown?	Depends on detail-level.
secs	**y**, **n**	Should side compounds be shown?	Depends on detail-level.
mstruct	**none**, **most**, **all**	Should compound structures for main compounds be shown? **most:** structures for common compounds, such as ATP and NAD, are omitted.	**none** or **most**, depending on detail-level.
sstruct	**none**, **most**, **all**	Should compound structures for side compounds be shown? **most:** structures for common compounds, such as ATP and NAD, are omitted.	**none** or **most**, depending on detail-level.
reglinks	**y**, **n**	If **n**, omit enzyme regulation icons and feedback inhibition links.	**y**
nolinks	**y**, **n**	If **y**, suppress showing links to other pathways.	**n**
pfontsize	**tiny**, **very-small**, **small**, **normal**, **large**, **very-large**	Font size used in diagram	**very-small**
bgcolor	**w**, **cb**, **g**, **bw**, **tr**	The color scheme for the diagram: **w**: Colors on a white background. **cb**: Colors on a black background. **g**: Colors on a gray background. **bw**: Black on a white background. **tr**: Same colors as for **w**, but on a transparent background.	**tr**
linear	**snake**, **horizontal**, **vertical**	Specify whether linear pathways are drawn horizontally, vertically or in a horizontal back-and-forth “snake”-like fashion. Only pathways that contain no cycles or branch points (including branch points resulting from links to/from other pathways) are recognized as linear pathways and, therefore, are sensitive to this parameter.	**snake**
